# Royal College of Surgeons

**Published:** 1909-04-10

**Authors:** 


### ROYAL COLLEGE OF SURGEONS.

At the last quarterly meeting of the Council of the Royal College of Surgeons of England on April 1, Sir Shirley Murphy, Medical Officer of Health to the County of London, and Mr. G. Dancer Thane, Professor of Anatomy at University College, London, were elected Fellows of the College under the clause of the Charter relating to the election of members of twenty years' standing. The Jacksonian Prize was awarded to Mr. J. P. Lockhart Mummery, F.R.C.S., for his essay on "The Pathology and Treatment of those Conditions and Diseases of the Colon which are Relievable by Operative Measures." The subject for next year will be "Tuberculous Disease of the Urinary Bladder and Male Genital Organs." The John Tomes Dental Prize for 1906-1908 was awarded to Mr. Arthur Swayne Underwood, M.R.C.S.,L.D.S.Eng. The By-law (in three sections) relating to the Admission of Women to Examination for the Diplomas and to their standing in the College was made and ordained, and the solicitor of the College was instructed to submit it to the government authorities for sanction and ratification. (The text of the new by-law was printed in this column on March 27, 1909, p. 678.) A letter was read from Mr. J. Ward Cousins reporting the proceedings of the Central Midwives Board during the past year, and a vote of thanks was accorded him for his very efficient services as the representative of the College on this Board during the past six years. Mr. C. H. Golding-Bird was elected as representative of the College in place of Mr. Ward Cousins, who has retired. Sir Jonathan Hutchinson was appointed the delegate of the College to the University of Geneva on the occasion of the celebration of the 350th anniversary of its foundation in July next. Mr. Pearce Gould's period of office on the Court of Examiners of the College will expire on May 12, and the vacancy will be filled up at the meeting of the Council on May 13. Mr. H. H. Clutton was re-elected one of the two representatives of the College on the Senate of the University of London, and a vote of thanks was given to Dr. Norman Moore for his services as visitor for the Royal Colleges of Physicians and Surgeons to the examinations of the Egyptian Medical School at Cairo. On the recommendation of the Museum Committee it was decided to ask Dr. R. T. Leiper, of the London School of Tropical Medicine, to undertake the revision and renovation of the collection of Entozoa in the College Museum.

